# Regulators of RNA m^5^C methylation are adipose tissue depot-specific expressed and correlate with clinical variables of obesity in humans

**DOI:** 10.3389/fendo.2025.1647477

**Published:** 2025-09-15

**Authors:** Stina Ingrid Alice Svensson, Anne Hoffmann, Tobias Hagemann, Akin Cayir, Sadia Saeed, Adhideb Ghosh, Christian Wolfrum, Matthias Blüher, Yvonne Böttcher

**Affiliations:** ^1^ Department of Clinical Molecular Biology, EpiGen, Institute of Clinical Medicine, University of Oslo, Oslo, Norway; ^2^ Helmholtz Institute for Metabolic, Obesity and Vascular Research (HI-MAG) of the Helmholtz Zentrum München at the University of Leipzig and University Hospital, Leipzig, Germany; ^3^ EpiGen, Medical Division, Akershus University Hospital, Lørenskog, Norway; ^4^ Laboratory of Translational Nutrition Biology, Institute of Food, Nutrition and Health, ETH Zürich, Schwerzenbach, Switzerland; ^5^ Medical Department III – Endocrinology, Nephrology, Rheumatology, University of Leipzig Medical Center, University of Leipzig, Leipzig, Germany

**Keywords:** adipose tissue, obesity, RNA methylation, epitranscriptomics, m^5^C regulators

## Abstract

**Background:**

Obesity is a global health burden and recent evidence indicates that epitranscriptomic regulation is potentially involved in its etiology. The epitranscriptomic mark 5-methylcytosine (m^5^C) is implicated in cancer and recent data linked the gene expression of m^5^C writers, erasers and readers to diabetes, a well-known co-morbidity of obesity. Here, we tested whether gene expression of m^5^C regulators in paired samples of human visceral and subcutaneous adipose tissue is **(i)** adipose tissue depot-specific and **(ii)** correlates with important clinical variables of obesity.

**Methods:**

Intra-individually paired adipose tissue samples from human subcutaneous adipose tissue (SAT) and omental visceral adipose tissue (OVAT) were utilized from three different cohorts from the Leipzig Obesity Biobank including a large cross-sectional cohort, a two-step bariatric surgery cohort and a cohort of metabolically healthy vs unhealthy individuals (LOBB, total *N=*962). Data analysis on intra-individual samples was performed by using the paired Wilcoxon signed-rank test, while in comparisons on independent groups the unpaired Wilcoxon rank-sum test was employed. Bonferroni correction method was used to adjust multiple testing of p-values and Spearman’s rank correlation was used to assess associations.

**Results:**

We observed that multiple m^5^C regulators were differentially expressed between human subcutaneous and visceral adipose tissue depots. Interestingly, we found that for several regulators the effects were less pronounced after weight loss, whilst stronger in individuals with insulin resistance compared to their healthy counterparts. A strong correlation of m^5^C regulator expression with macrophages was observed in OVAT compared to its SAT counterpart. Correlations between m^5^C regulators with important clinical variables related to obesity were observed in all three cohorts.

**Conclusion:**

Our findings provide evidence for adipose tissue depot-specific gene expression of m^5^C regulators that correlate with clinical variables of obesity.

## Introduction

Obesity is a global health burden and the World Health Organization has predicted that 46% of the worlds adult population will be affected by obesity in 2025 (https://data.worldobesity.org/publications/?cat=22). It is well known that especially accumulation of fat in visceral depots rather than subcutaneous counterparts entails a higher risk of metabolic disorders and co-morbidities (1–3). There have been multiple studies reporting transcriptional and epigenetic differences between human subcutaneous adipose tissue (SAT) and omental visceral adipose tissue (OVAT), along with recent evidence for depot-specific differential chromatin landscapes showing that OVAT is more open and accessible compared to SAT (4–8). Moreover, multiple clinical variables related to obesity and type 2 diabetes such as body mass index (BMI), waist-to-hip ratio (WHR), fasting plasma glucose (FPG), glycosylated hemoglobin (HbA1c), homeostasis model assessment of insulin resistance (HOMA-IR) and triglyceride levels correlate with gene expression and epigenetic marks in adipose tissue (9–11).However, the exact molecular mechanisms leading to a higher risk for metabolic complications are not yet fully understood.

A growing area of interest is the understanding of post-transcriptional RNA modifications in metabolic diseases (12, 13). The best studied post-transcriptional modification on messenger RNA (mRNA) is m^6^A (N^6^-methyladenosine) that may impact on mRNA metabolism by mRNA decay, pre-mRNA processing, export and translation (14). There is evidence that gene expression of m^6^A modifiers is adipose tissue depot-specific and correlates with clinical variables related to obesity (7). Another RNA modification of high interest is m^5^C (5-methylcytosine) that was shown to have more than 10,000 potential sites for modification in the human transcriptome, including transfer RNA (tRNA), ribosomal RNA (rRNA), mRNA and non-coding RNAs (ncRNAs) (15).

m^5^C is a dynamic epitranscriptomic mark that is regulated by protein groups important for installing m^5^C methylation (methyltransferases- writers), removing m^5^C marks by demethylation (erasers) and for functional translation by binding proteins (readers) (14). m^5^C regulation has reportedly been linked to changes in gene expression, RNA stability and protein synthesis, impacting both cellular and physical processes (15).

A recent report described an association of m^5^C-regulators with type 2 diabetes mellitus (T2D) (16). Through an integrated analysis of gene expression, Song et al. (2022) showed an enrichment of GO (Gene Ontology) and KEGG (Kyoto Encyclopedia of Genes and Genomes) pathways that were closely related to metabolism-related biological processes (16). The following genes encoding m^5^C regulators were investigated by Song et al. (2022): 3 erasers (*TET1, TET2*, and *TET3*), 11 writers (*DNMT1, DNMT2, DNMT3A, DNMT3B, NSUN1, NSUN2, NSUN3, NSUN4, NSUN5, NSUN6* and *NSUN7*) and 16 readers (*ALYREF, MBD1, MBD2, MBD3, MBD4, MECP2, NEIL1, NTHL1, SMUG1, TDG, UHRF1, UHRF2, UNG, YBX1, ZBTB4*, and *ZBTB33*). The list of genes was generated by Song et al. (2022) through a review of the literature and based on publications from Du et al. (2020) and Zhang et al. (2021) (16–18). The authors observed that gene expression of m^5^C regulators significantly correlated with genes involved with T2D (16).

Additionally, recent studies support a role of m^5^C in adipose tissue biology. Liu et al. (2021) showed that the knockdown of *NSUN2* (NOL1/NOP2/Sun domain family member 2), a well-known m^5^C writer, increased lipid accumulation in 3T3-L1 pre-adipocytes by interacting with *CDKN1A* (cyclin-dependent kinase inhibitor 1A) in early stages of adipogenesis (19). Further, Liu et al. (2022) discovered in adipocytes from pigs that the m^5^C reader ALYREF (Aly/REF export factor) was involved in recognizing and transporting *YBX2* (Y-box binding protein 2) and *SMO* (smoothened, frizzled class receptor) into the cytoplasm, leading to inhibited adipogenesis and increased myogenesis (20).

Inspired by these studies, we set out to better understand the role of m^5^C regulators in human obesity with a special emphasis on gene expression in different human adipose tissue depots (SAT vs. OVAT). To our knowledge, no studies exist exploring transcription of the m^5^C machinery in distinct adipose tissue depots and evaluating its relationship to clinical variables of obesity. To do so, we utilized the m^5^C regulator gene list as described in Song et al. (2022) and applied it to intra-individually paired samples of SAT and OVAT from the LOBB (Leipzig Obesity Biobank) cohort (21, 22). We aimed to **(i)** test whether gene expression of the m^5^C regulators is adipose tissue depot-specific; and **(ii)** whether gene expression of these genes correlates with clinical variables of obesity.

## Method and materials

### Cohort description

In this study, we used the Leipzig Obesity BioBank (LOBB; https://www.helmholtz-munich.de/en/hi-mag/cohort/leipzig-obesity-bio-bank-lobb)comprising intra-individually paired SAT and OVAT samples organized in several sub cohorts (total *N*=962). All patients were extensively clinically phenotyped regarding obesity, metabolic variables and anthropometric traits as described by Blüher (2020) and Klöting et al. (2010) (21, 22). Adipose tissue samples were collected during elective laparoscopic abdominal surgeries as described previously (23). Exclusion criteria comprised individuals under the age of 18, chronic substance or alcohol misuse, smoking within the last year leading up to surgery, acute inflammatory diseases, end-stage malignant diseases, uncontrolled thyroid disorder, and Cushing’s disease. Three sub cohorts of LOBB were considered: a cross-sectional cohort (CSC, *N*=839), a two-step bariatric surgery cohort (BSC, *N*=52) and a cohort comprising individuals with metabolically healthy vs. unhealthy obesity (MHUO, *N*=71). The CSC cohort comprises 818 subjects with obesity (72% women, 40% T2D, mean ± sd age 46.9 ± 11.8 years; mean ± sd BMI 48.9 ± 8 kg/m^2^ *)* and 21 subjects without obesity (62% women, 17% T2D, mean ± sd age 49 ± 13.5 years; mean ± sd BMI 27 ± 2.5 kg/m^2^). The BSC cohort consists of 52 individuals that have undergone a two-step bariatric surgery at two time points and who were considered morbid obese at the first surgery. Only individuals who lost more than 10 kg of weight were considered. The first time point (first surgery) is labelled pre-surgery (70% women, 58% T2D, mean ± sd age 44.5 ± 9.6 years; mean ± sd BMI 54.3 ± 10.6 kg/m^2^), the second time point (second surgery) is labelled post-surgery (50% T2D, mean ± sd age 46.7 ± 9.6 years; mean ± sd BMI 40.2 ± 7.5 kg/m^2^). Individuals lost 41.1 ± 21.2 kg between the two surgeries. The MHUO cohort entails 30 obese individuals who are insulin sensitive (IS; 71% women, mean ± sd glucose infusion rate (GIR) clamp 89.5 ± 9.3 mmol/kg/min, mean ± sd age 38.5 ± 11.4 years; mean ± sd BMI 44.5 ± 7.1 kg/m^2^, 10% T2D) and 41 individuals who are insulin resistant (IR; 73% women, mean ± sd GIR clamp 28.7 ± 12–6 mmol/kg/min, mean ± sd age 50.4 ± 8 years; mean ± sd BMI 47.2 ± 6.9 kg/m^2^) 61% T2D). The cohort participants were stratified into metabolically healthy and unhealthy obesity groups according to previously published parameters. A detailed description of the MHUO cohort is provided in Reinisch et al., 2025 (31).

### Library preparation and RNA-seq analysis

Library preparation and RNA sequencing (RNA-seq) were carried out as previously described (24). Briefly, RNA was isolated from adipose tissue samples using the SMARTseq (Switch Mechanisms at the 5’ End of RNA Templates Sequencing) protocol (25). Sequencing libraries were generated and subjected to single-end sequencing on a NovaSeq 6000 platform at the Functional Genomics Centre Zurich.

Raw sequencing reads were processed using Fastp (v0.23.4) for adapter removal and quality trimming (26). A sliding window approach was applied, trimming the read ends when the average quality within a four-base window dropped below a Phred quality score of 20. Reads shorter than 18 bases after trimming were discarded. Trimmed reads were aligned to the human reference genome (GRCh38, GENCODE release 47) using STAR (v2.7.11b), allowing up to 50 multiple alignments per read (27). Gene-level quantification was performed using featureCounts (v1.5.3), with fractional assignment for reads mapping to multiple genomic locations (28). Count data were transformed using variance-stabilizing transformation (VST) via the DESeq2 R package v1.48.1, and expression values were adjusted for age, sex and proportion of reads assigned to features (exon mapping rate) using the r package limma v 3.64.0 before further analysis (29, 30).

### Statistical analyses

Analysis was conducted in R 4.5.0. Statistical significance was assessed using Wilcoxon tests implemented via the rstatix R package (v0.7.2). For comparisons involving intra-individually paired samples (pre- and post-intervention measures from the same individual; tissue comparisons), the paired Wilcoxon signed-rank test was applied. For comparisons between independent groups, the unpaired Wilcoxon rank-sum test was used. P-values were adjusted for multiple testing using the Bonferroni correction method. Spearman’s rank correlation coefficients were calculated to assess monotonic associations between variables. Confidence intervals for the correlation coefficients were computed using the standard Fisher z-transformation. All correlation analyses were performed using the RVAideMemoire R package (v0.9-83-11).

## Results

### Obesity- and depot-specific regulation of m^5^C regulators in adipose tissue

**Figure 1:**
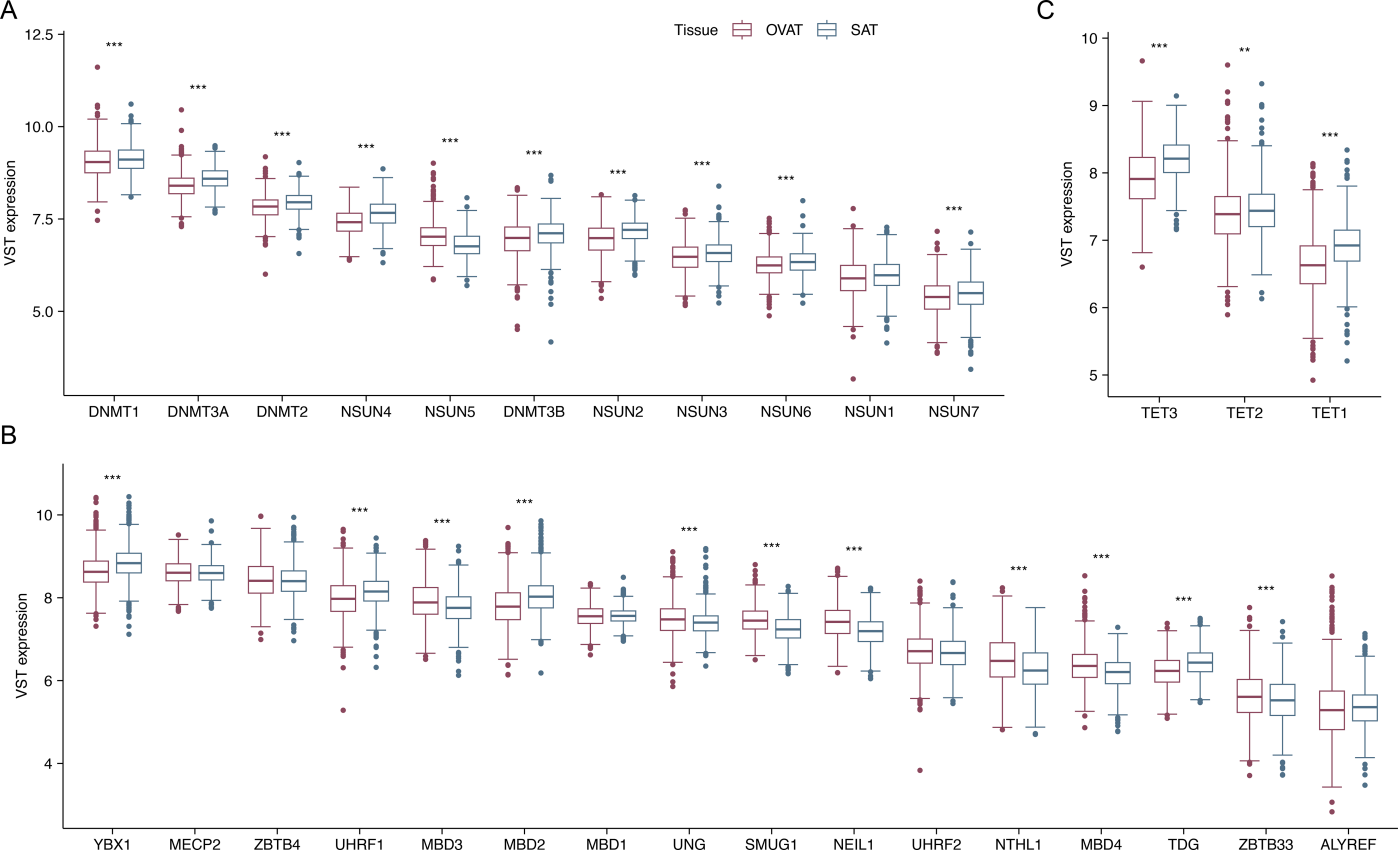
Differential gene expression of m^5^C regulators in intra-individually paired samples from subcutaneous and omental visceral adipose tissue in individuals from the cross-sectional cohort. Gene expression levels of all selected **(A)** m^5^C writers, **(B)** m^5^C readers and **(C)** m^5^C erasers are compared between subcutaneous adipose tissue (SAT; blue) and omental visceral adipose tissue (OVAT; red) within the same obese individuals (N=818) of the cross-sectional cohort (CSC). Statistical significance was assessed using paired Wilcoxon tests, with p-values corrected for multiple comparisons via Bonferroni correction. P value legend, <0.001 (***), <0.01 (**), <0.05 (*). VST, variance stabilizing transformation.

To assess whether genes encoding for the m^5^C regulatory machinery are expressed in an adipose tissue depot-specific manner, we evaluated whether their expression levels differ significantly among our individuals with obesity of the cross-sectional cohort (*N*=818). Multiple writers (*DNMT1*, *DNMT3A*, *DNMT3B*, *DNMT2*, *NSUN2*, *NSUN3*, *NSUN4*, *NSUN5*, *NSUN6*, and *NSUN7*; all adj. p < 0.001; **Figure 1A**), readers (*MBD2*, *MBD3*, *MBD4*, *NEIL1*, *NTHL1*, *SMUG1*, *TDG*, *UHRF1*, *UNG*, *YBX1*, and *ZBTB33*; all adj. p<0.001; **Figure 1B**) and all of the known erasers (*TET1*, *TET2*, and *TET3;* all adj. p<0.01; **Figure 1C**) are indeed significantly different expressed in SAT vs. OVAT (**Supplementary Table 1**). Except *NSUN5*, the writers had a significantly lower expression in OVAT compared to SAT. This may hint at a potentially decreased deposition of the m^5^C mark on RNA molecules in OVAT. Moreover, in contrast, most of the genes encoding for reader proteins (*MBD3*, *UNG*, *SMUG1*, *NEIL1*, *NTHL1*, *MBD4*, and *ZBZB33*), which are generally important for RNA metabolism and function, were significantly upregulated in OVAT. The three erasers removing m^5^C from RNA molecules were downregulated in OVAT compared to SAT.

Furthermore, in OVAT, the *DNMT3A* writer gene (adj. p < 0.001) and the eraser enzymes *TET3* and *TET2* (adj. p<0.01) were significantly downregulated in individuals with obesity (*N*=818) compared to non-obese controls (*N*=21). Conversely, several reader genes (*MBD2*, *SMUG1*, and *MBD4*) were upregulated (adj. p < 0.05) in individuals with obesity (**Supplementary Figure 1**; **Supplementary Table 1**). In SAT, the eraser enzymes *TET1* and *TET2* (adj. p<0.001) were downregulated in obese individuals, along with the reader *UHRF2* and the writer *NSUN3* (adj. p<0.01). The writer *DNMT1* (adj. p<0.01) and the reader *MBD3* (adj. p<0.05) were upregulated in obese individuals (**Supplementary Figure 2**; **Supplementary Table 1**).

Taken together, these data from our large cross-sectional cohort provide sound evidence for adipose tissue depot-specific gene expression of m^5^C regulating genes. Some regulatory genes are differentially expressed between non-obese subjects and individuals with obesity, while certain patterns such as decreased eraser activity among obese individuals are observed across multiple depots.

### m^5^C regulators correlate with fat accumulation, adipocyte size and metabolic traits in a depot-specific manner

**Figure 2:**
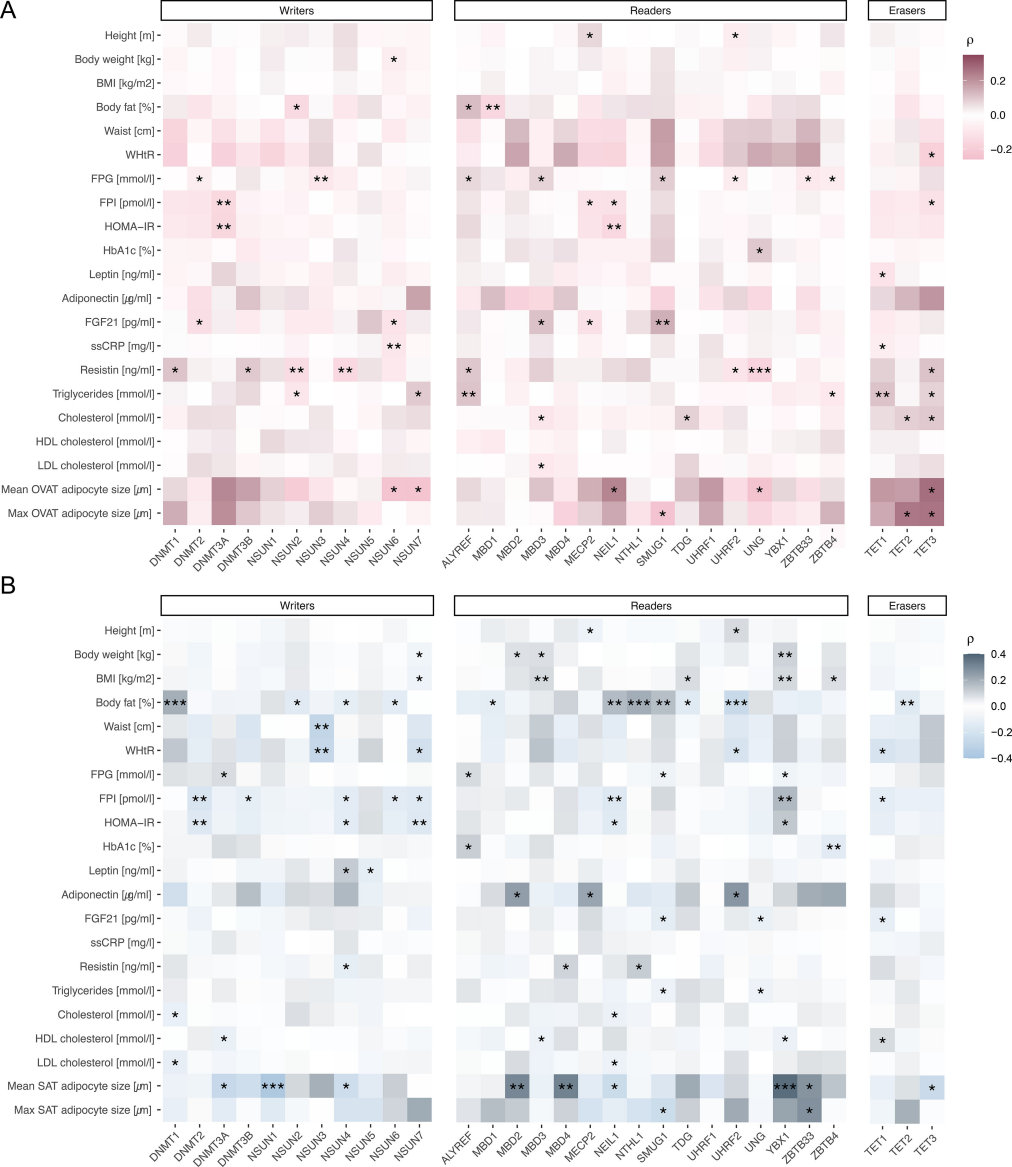
m^5^C regulators correlates with clinical variables of obesity in the CSC cohort. Spearman correlations of all selected m5C regulators included m^5^C writers, m^5^C readers and m^5^C erasers with obesity-related clinical variables are shown for **(A)** OVAT and **(B)** SAT. The color scheme representing the strength and direction of the correlation. P value (not adjusted for multiple testing) legend, <0.001 (***), <0.01 (**), <0.05 (*). BMI, body mass index; EBMIL, excess body mass index loss; FPG, fasting plasma glucose; FPI, fasting plasma insulin; FGF21, fibroblast growth factor 21; HOMA-IR, homeostatic model assessment of insulin resistance; HDL cholesterol, high-density lipoprotein cholesterol; HbA1c, Hemoglobin A1c; LDL cholesterol, low-density lipoprotein cholesterol; ssCRP, serum soluble C-reactive protein; VST, variance stabilizing transformation; WHtR, waist-to-height ratio.

We observed multiple correlations (p<0.05) between the expression levels of m^5^C methylation regulators and clinical variables related to obesity within the cross-sectional cohort (**Figure 2**, **Supplementary Table 2**). Most of these correlations with anthropometric variables were identified for body fat (*N*=292). In OVAT, *NSUN2* (writer, ρ=-0.15, p=0.012) and *MBD1* (reader, ρ=-0.16, p=0.007) exhibited weak negative correlations, while *ALYREF* (reader, ρ=0.12, p=0.041) showed a positive correlation with body fat (**Figure 2A**). In contrast, the SAT samples showed stronger negative correlations between body fat and several methylation regulators, including the writers *NSUN2* (ρ=-0.14, p=0.016), *NSUN4* (ρ=-0.12, p=0.048), and *NSUN6* (ρ=-0.13, p=0.027), as well as the readers *UHRF2* (ρ=-0.26, p<0.001), *TDG* (ρ=-0.14, p=0.014), and *MBD1* (ρ=-0.13, p=0.028), along with the eraser *TET2* (ρ=-0.15, p=0.009). Positive correlations with body fat were observed for the writer *DNMT1* (ρ=0.2, p<0.001) and the readers *SMUG1* (ρ=0.16, p=0.007), *NEIL1* (ρ=0.17, p=0.005), and *NTHL1* (ρ=0.2, p<0.001) (**Figure 2B**).

Additionally, positive correlations were found between maximum adipocyte size in OVAT (*N*=75) and the erasers *TET2* (ρ=0.26, p=0.027) and *TET3* (ρ=0.26, p=0.024) suggesting that higher activity of these enzymes may be associated with larger fat cells. Conversely, *SMUG1* showed a negative correlation (ρ=-0.24, p=0.041), indicating that increased *SMUG1* expression might be linked to smaller adipocytes (**Figure 2A**). In SAT, *ZBTB33* was positively correlated (ρ=0.28, p=0.016), while *SMUG1* remained negatively correlated (ρ=-0.24, p=0.037) with the maximum adipocyte size (*N*=75) (**Figure 2B**).

In both adipose tissues, associations between metabolic parameters and m^5^C methylation regulators were observed. For example, in OVAT, *DNMT3A* (ρ=-0.16, p=0.007) and *NEIL1* (ρ=-0.15, p=0.009) showed negative correlations with HOMA-IR (*N*=288), suggesting that higher activity of these genes may be linked to improved insulin sensitivity. Several methylation regulators also demonstrated correlations with circulating FGF21 (fibroblast growth factor 21) levels (*N*= 360). *MBD3* (ρ=0.11, p=0.033) and *SMUG1* (ρ=0.15, p=0.006) were positively associated with FGF21 levels, while *DNMT2* (ρ=-0.13, p=0.014), *MECP2* (ρ=-0.12, p=0.024), and *NSUN6* (ρ=-0.11, p=0.034) exhibited weak negative correlations. These findings imply a potential epigenetic influence on FGF21 regulation, which could impact metabolic signaling pathways. Furthermore, resistin levels (*N*=420) were inversely correlated with several methylation regulators namely *UHRF2* (ρ=-0.11, p=0.027), *UNG* (ρ=-0.17, p<0.001), *NSUN2* (ρ=-0.14, p=0.004), and *NSUN4* (ρ=-0.15, p=0.002), as well as positively associated with *ALYREF* (ρ=0.1, p=0.036), *DNMT1* (ρ=0.11, p=0.028), *DNMT3B* (ρ=0.1, p=0.049), and *TET3* (ρ=0.11, p=0.026) suggesting these enzymes may have a role in inflammation or adipokine regulation (**Figure 2A**). In SAT, several methylation regulators were also associated with HOMA-IR, including *DNMT2* (ρ=-0.16, p=0.008), *NEIL1* (ρ=-0.15, p=0.014), *NSUN4* (ρ=-0.13, p=0.028), *NSUN7* (ρ=-0.16, p=0.008), and *YBX1* (ρ=0.14, p=0.022; *N*=288). Additionally, *ALYREF* (ρ=0.12, p=0.012) and *ZBTB4* (ρ=-0.13, p=0.004) showed associations with HbA1c (*N*=450). Positive correlations with adiponectin levels were observed exclusively in SAT and involved *MECP2* (ρ=0.22, p=0.047), *MBD2* (ρ=0.24, p=0.031), and *UHRF2* (ρ=0.26, p=0.018) (**Figure 2B**).

In summary, these findings highlight the potential role of specific m^5^C regulators in fat accumulation, adipocyte size, and obesity-related traits in a depot-specific manner, providing insights into potential epigenetic contributions to obesity and metabolic health.

### Depot-specific adipose tissue gene expression of m^5^C regulators is less pronounced in the two -step bariatric surgery cohort

**Figure 3:**
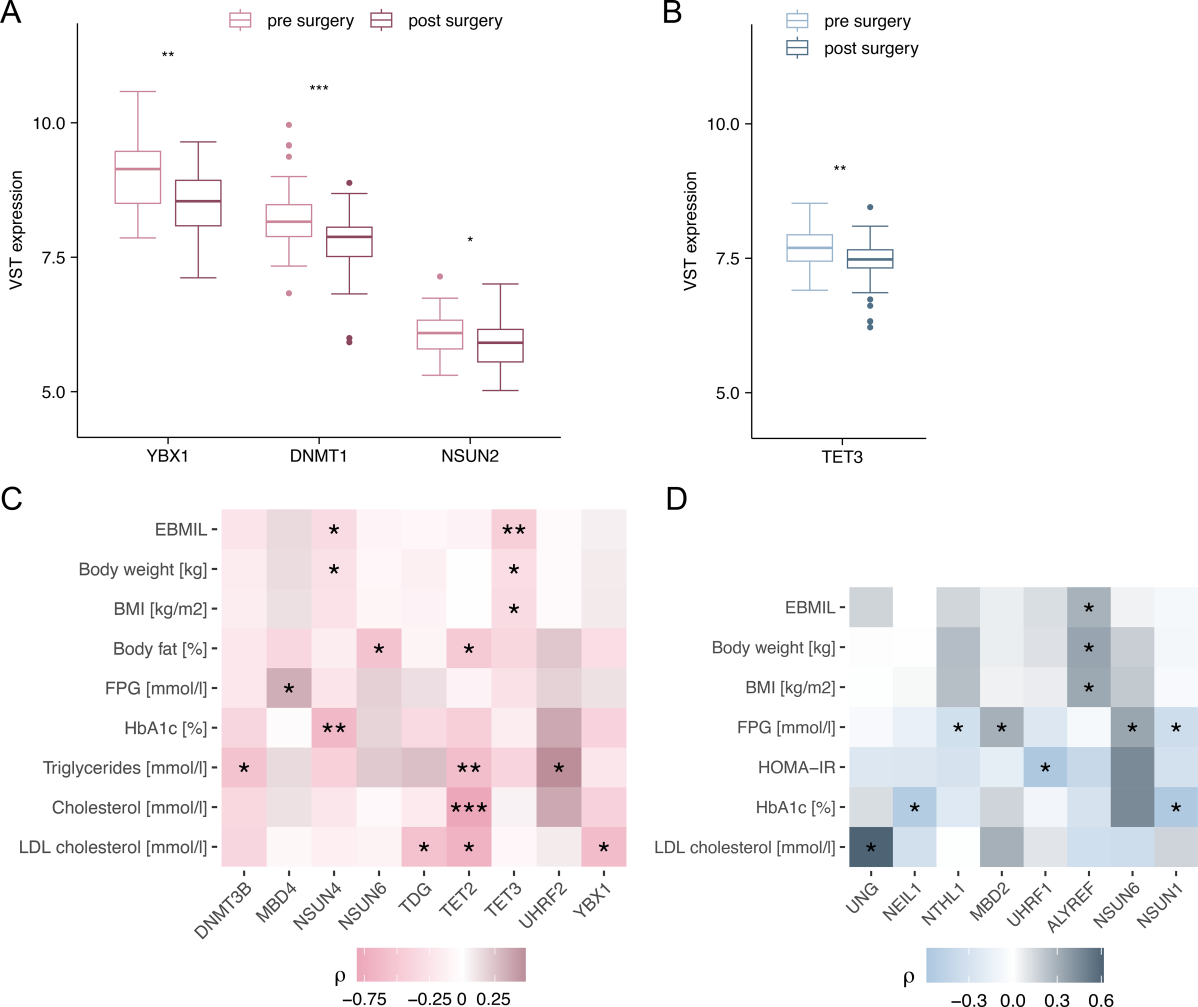
m^5^C regulator expression differences and their clinical correlations in intra-individually paired samples from SAT and OVAT in individuals following weight loss. Only the gene expression comparisons of m^5^C regulators that showed significant differences between pre- and post-surgery expression levels are presented for **(A)** OVAT and **(B)** SAT within the same individuals (N=52) of the bariatric surgery cohort. Statistical significance was assessed using paired Wilcoxon tests, with p-values corrected for multiple comparisons via Bonferroni correction. Spearman correlations (unadjusted p<0.05 only) between the delta (pre-post) expression levels of m^5^C regulators and the delta of clinical parameters are illustrated for **(C)** OVAT and **(D)** SAT. The color scheme representing the strength and direction of the correlation. P value legend, <0.001 (***), <0.01 (**), <0.05 (*). BMI, body mass index; EBMIL, excess body mass index loss; HbA1c, Hemoglobin A1c; FPG, fasting plasma glucose; LDL cholesterol, low-density lipoprotein cholesterol; VST, variance stabilizing transformation.

Next, we tested whether transcriptional levels of the m^5^C regulatory machinery are affected by weight loss in the two-step bariatric surgery cohort and whether this relates to any correlation with clinical parameters. We found significant (adj. p < 0.05) differences in gene expression of four m^5^C regulators between the pre- and post-surgery timepoints (*DNMT1*, *NSUN2*, and *YBX1* in OVAT, **Figure 3A**; *TET3* in SAT, **Figure 3B**). All these regulators exhibited significantly lower expression levels after weight loss (post-surgery) compared to baseline (pre-surgery) values. An overview of all comparisons is provided in **Supplementary Table 3**. No other m^5^C regulators were found differentially expressed.

The correlation analysis between delta (pre – post) changes in m^5^C regulator expression and delta weight loss-related phenotypes in our bariatric cohort revealed several associations (p<0.05) (**Supplementary Table 4**, **Figures 3C, D**). In OVAT, several correlations were observed between the expression changes of several m^5^C regulators and measures of fat loss or lipid-related phenotypes. Specifically, decreases in *NSUN4* (ρ=-0.33, p=0.018, *N*=52) and *TET3* (ρ=-0.44, p=0.001, *N*=52) delta expression values were associated with greater reductions in EBMIL (excess body mass index loss), indicating that lower *NSUN4* and *TET3* levels post-surgery may be linked to more effective weight loss. Decreases in *NSUN6* (ρ=-0.55, p=0.025, *N*=17) and *TET2* (ρ=-0.49, p=0.049, *N*=17) expressions were associated with reductions in body fat. In addition, strong inverse correlations were observed between *TET2* (ρ=-0.69, p=0.011, *N*=13), *TDG* (ρ=-0.56, p<0.05, *N*=13), and *YBX1* (ρ=-0.62, p=0.029, *N*=13) expression and LDL (low-density lipoprotein) cholesterol. Regarding triglycerides, decreases in *DNMT3B* (ρ=-0.55, p=0.016, *N*=19) and *TET2* (ρ=-0.6, p=0.008, *N*=19) delta expressions were linked to lower delta triglyceride levels. Conversely, an increase in *UHRF2* (ρ=0.48, p=0.037, *N*=19) expression was positively correlated with triglyceride levels (**Figure 3C**).

In SAT, changes in *ALYREF* expression were positively correlated with measures of fat loss, including BMI (ρ=0.33, p=0.016, *N*=52), body weight (ρ=0.35, p=0.011, *N*=52), and EBMIL (ρ=0.29, p=0.036, *N*=52). These results suggest that increased *ALYREF* expression in SAT may be associated with greater reductions in body mass and enhanced metabolic activity during weight loss. In contrast to OVAT, the expression levels of several m^5^C regulators in SAT were associated with FPG and HOMA-IR. Specifically, *NSUN1* (ρ=-0.36, p=0.016, *N*=44) and *NTHL1* (ρ=-0.33, p=0.029, *N*=44) expression showed a negative correlation with FPG, indicating that higher *NSUN1* and *NTHL1* levels may be linked to lower FPG. Conversely, *NSUN6* (ρ=0.35, p=0.022, *N*=44) and *MBD2* (ρ=0.3, p=0.048, *N*=44) expression levels show a positive correlation with FPG. Further, *UHRF1* expression showed a strong negative correlation (ρ=-0.59, p=0.049, *N*=12) with HOMA-IR, implying that higher *UHRF1* levels might be associated with improved insulin sensitivity in SAT (**Figure 3D**).

This data illustrates that the gene expression profile of the m^5^C regulatory machinery is altered in the two-step bariatric surgery group and that such changes seem to be related to metabolic parameters. This inspired us to next evaluate the expression profiles in a cohort comprising metabolically healthy vs. unhealthy individuals with obesity.

### Cell type–resolved m^5^C regulator profiles suggest stromal and immune involvement in IR-associated adipose tissue changes

**Figure 4:**
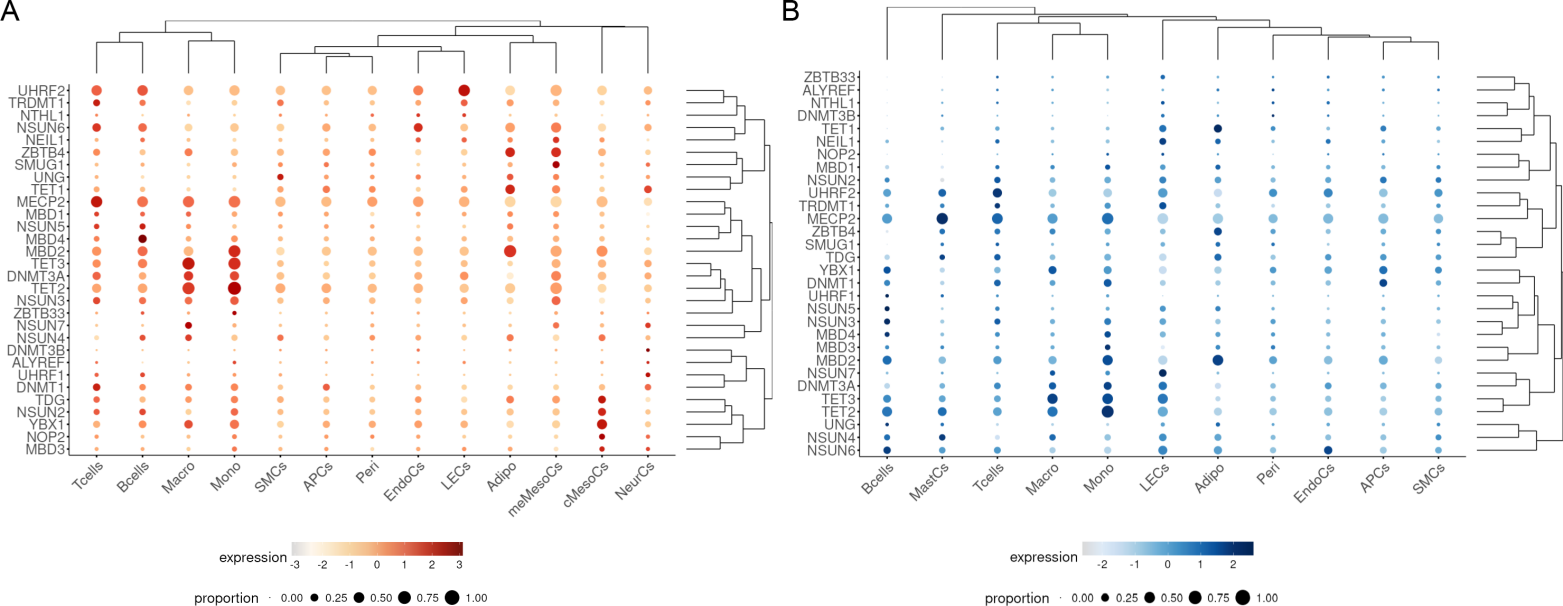
Expression levels of m^5^C regulators across different cell types in single-nucleus RNA sequencing data from individuals with (insulin sensitivity) IS and (insulin resistance) IR. The dot plots display the expression intensity of m^5^C regulators, differentiated by cell type in **(A)** OVAT and **(B)** VAT of individuals with IS and IR of the metabolically healthy vs. unhealthy obesity cohort. Expression levels are indicated by color intensity, while the proportion of cells expressing each gene is represented by the size of the dots. Adipo, adipocytes; APCs, antigen-presenting cells; cMesoCs, classical mesothelial cells; EndoCs, endothelial cells; LECs, lymphatic endothelial cells; Macro, macrophages; MastCs, mast cells; meMesoCs, mesenchymal mesothelial cells; Mono, monocytes; NeurCs, neuronal cells; SMCs, smooth muscle cells; Peri, pericytes. Note that some genes are shown under their alternative names, *NOP2* is listed as *NSUN1*, and *TRDMT1* corresponds to *DNMT2*. The data were analyzed using the web applications available at https://github.com/WolfrumLab/MHUO/.

To profile the expression landscape of m^5^C regulatory genes across various cell types, single-nucleus RNA sequencing (snucSeq) data from IS and IR morbidly obese individuals were analyzed (**Figure 4**) (31). These data were obtained from Reinisch et al., 2025 (31), and include a subset of participants from our metabolically healthy versus unhealthy obesity (MHUO) cohort. In brief, snucSeq was performed on pooled adipose tissue samples from metabolically healthy and unhealthy obese individuals, comprising 41 OVAT and 36 SAT samples. Despite an overall lower gene expression of m^5^C regulators in SAT compared to OVAT, several m^5^C regulator genes displayed markedly elevated expression levels and were broadly distributed across various immune cell types (e.g., macrophages, monocytes, and T cells) and stromal cell types (e.g., adipocytes and mesothelial cells). Specifically, *MECP2*, *MBD2*, *TET3*, *TET2*, and *DNMT3A* were strongly expressed in macrophages, a key immune cell type involved in adipose tissue inflammation and metabolic dysfunction. These genes also showed high expression in monocytes, suggesting their broader involvement in innate immune regulation. Among the regulators, *MECP2* exhibited the strongest and most widespread expression across virtually all cell types, suggesting a potentially global role in transcriptional and epigenetic regulation within adipose tissue. *MBD2* showed the highest expression in adipocytes in both tissues, as well as in meMesoCs (mesenchymal-like mesothelial cells) and cMesoCs (mesothelial cell subtypes) in OVAT, indicating a possible role in modulating both lipid storage and mesothelial cell identity or function. *TET1* and *ZBTB4* are also stronger expressed in adipocytes (**Figure 4**).

**Figure 5:**
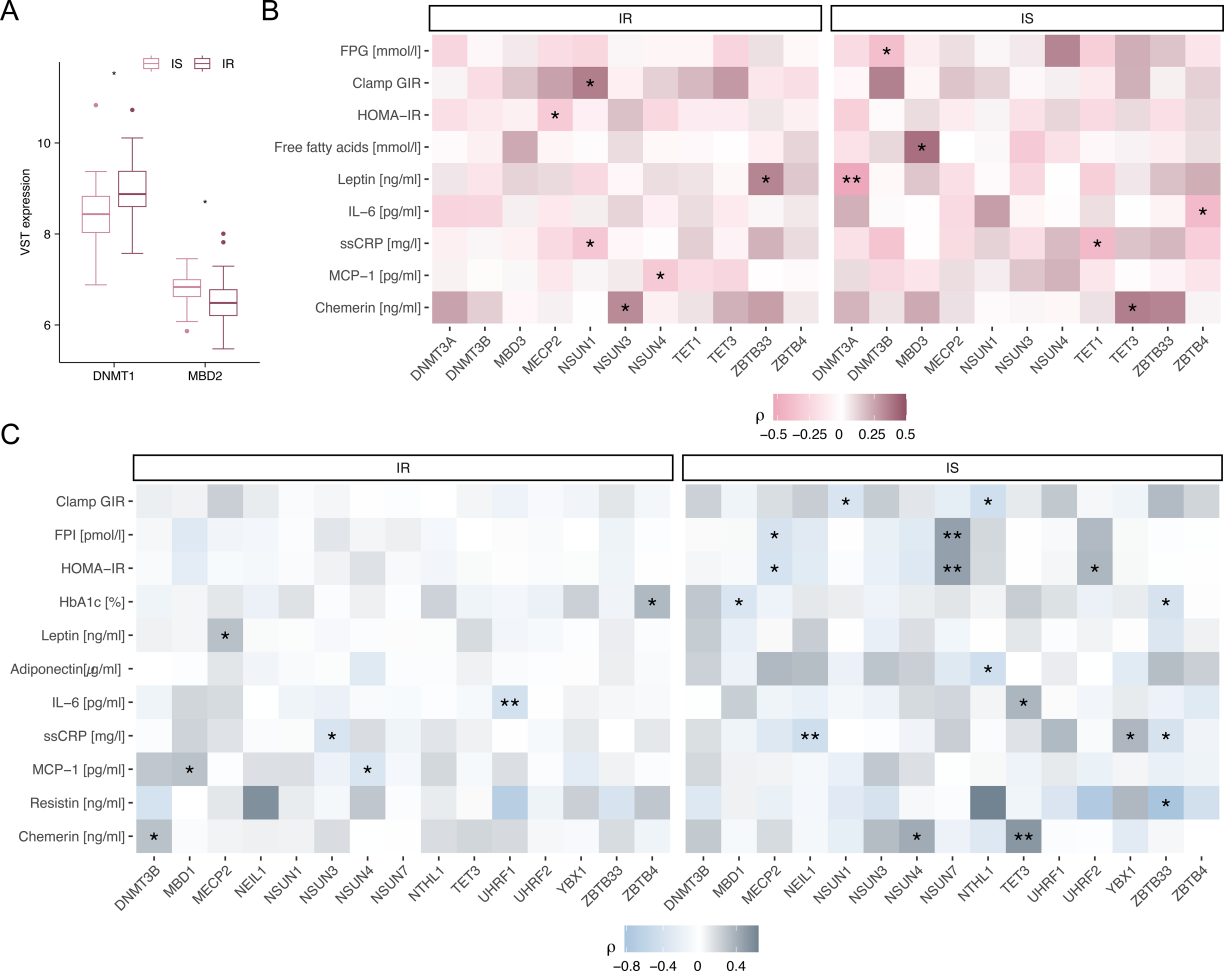
m^5^C regulator expression differences and their clinical correlations in intra-individually paired samples from SAT and OVAT in individuals considered having healthy or unhealthy metabolic traits. Only the gene expression comparisons of m^5^C regulators that showed significant differences between expression levels of individuals which are insulin sensitive (IS; N=30) and insulin resistant (IR; N=41) are presented for **(A)** OVAT of the metabolically healthy vs. unhealthy obesity cohort. Statistical significance was assessed using paired Wilcoxon tests, with p-values corrected for multiple comparisons via Bonferroni correction. Spearman correlations (unadjusted p<0.05 only) for IS and IR expression levels of m^5^C regulators and clinical parameters are illustrated for **(B)** OVAT and **(C)** SAT. The color scheme representing the strength and direction of the correlation. P value legend, <0.01 (**), <0.05 (*). FPG, fasting plasma glucose; FPI, fasting plasma insulin; GIR, glucose infusion rate; HbA1c, Hemoglobin A1c; HOMA-IR, homeostatic model assessment of insulin resistance; IL-6, interleukin 6; MCP-1, monocyte chemoattractant protein 1; ssCRP, serum soluble C-reactive protein; VST, variance stabilizing transformation.

Analyses of m^5^C regulator RNAseq expression values between IS and IR samples of the MHUO cohort further (**Figure 5**, **Supplementary Tables 5**, **6**) supported the functional relevance of selected m^5^C regulators identified by snucSeq. For example, *MBD2* expression is significantly reduced in omental adipose tissue of IR individuals compared to IS individuals (**Figure 5A**), suggesting a potential loss of epigenetic regulation in key stromal cell types associated with insulin resistance. In IS individuals OVAT, *DNMT3A* expression is significantly negatively correlated with circulating leptin levels (ρ=-0.52, FDR=0.003), suggesting a potential anti-inflammatory or metabolically protective role. Additionally, *TET3* expression in IS individuals in both tissues positively correlated with chemerin levels (OVAT: ρ=0.34, p=0.049; SAT: ρ=0.54, p=0.003) and positively with IL-6 levels in SAT (ρ=0.37, p=0.043) consistent with its stromal expression and involvement in adipokine regulation. Correlations observed for *MECP2* and *ZBTB4* in both IS and IR individuals, further support their roles in glycemic and inflammatory control, in line with their expression across adipocytes and immune cell types (**Figure 5B**).

## Discussion

Epitranscriptomic regulation in human adipose tissue through m^5^C methylation and its potential role in obesity is so far not well and systematically investigated. This work was inspired by the previous publication by Song et al. (2022) that shortlisted and identified multiple m^5^C regulators being related to type 2 diabetes through in-silico analyses (16). Here, we report that **(i)** m^5^C regulators are expressed in an adipose tissue depot-specific manner; **(ii)** and that gene expression levels of m^5^C regulators correlate with important clinical variables of obesity.

### m^5^C regulators are adipose tissue depot-specific expressed

The role of the epitranscriptomic mark m^5^C has not been previously well described in the aetiology of obesity. A handful of studies reported a potential role of m^5^C in adipogenesis. Liu et al. (2021) provided insights into the molecular function of mRNA m^5^C in relation to the export of *CDKN1A* mRNA from the nucleus in controlling adipogenesis (19). They report the direct involvement of the writer and methyltransferase *NSUN2* in regulating the m^5^C levels and the reader protein *ALYREF* to export *CDKN1A* mRNA to the cytoplasm (19). Furthermore, *ALYREF* was demonstrated to export *YBX2* and *SMO* mRNA into the cytoplasm of porcine cells in a m^5^C-dependent manner to inhibit adipogenesis and promote myogenesis respectively (20). Hence, the findings reported by Liu et al. (2021) illustrate that known m^5^C regulators are involved in controlling adipogenesis, suggesting a possible link with obesity (19, 20). Our results provide novel insights in the gene expression profiles of writers, readers and erasers of m^5^C methylation in human adipose tissue and about depot-specific expression patterns in SAT vs. OVAT along with its adipose residing cell types in individuals with obesity, after weight loss and in individuals with metabolic alterations. In general, all except for *NSUN5* of the m^5^C writers included in our study showed consistently lower gene expression in OVAT compared to SAT in the large cross-sectional cohort. The higher gene expression for m^5^C writers in SAT, especially the *NSUN* family with *NSUN2, NSUN3, NSUN4, NSUN6* and *NSUN7* all providing strong significances (p <0.001), is very interesting as higher levels of *NSUN2* have been shown to inhibit adipogenesis (19, 20). This may indicate that depot-specific alterations in adipogenesis governed by m^5^C may be related to metabolic and anthropometric variables. Moreover, the writer *NSUN3* was reported to catalyze the formation of m^5^C in mitochondrial tRNAs. (13). Given that mitochondrial dysfunction is known to be a consequence of obesity (32, 33), depot-specific *NSUN3* gene expression may indicate at mitochondrial m^5^C potentially involved in obesity in a depot-specific manner (13). Moreover, one can speculate that a potentially higher activity of the writing machinery in SAT compared to OVAT may potentially lead to lower m^5^C deposition in OVAT. However, since our study lacks functional validation, more studies are warranted to test this. No causative conclusions can be drawn so far and the data presented in this study should therefore be interpreted with caution.

Moreover, our data indicate that weight loss (as demonstrated in our two-step bariatric surgery cohort) seems to be related to less pronounced depot-specific differences between SAT and OVAT. Four m^5^C regulators *YBX1, DNMT1, NSUN2* in OVAT and *TET3* in SAT, are differentially expressed between pre- and post-surgery, and their expression levels were all downregulated following weight loss. Interestingly, when analyzing cell-type specific gene expression by using snucSeq data, we discovered several genes strongly expressed in immune cells. The elevated expression of *MECP2*, *MBD2*, *TET3*, *TET2*, and *DNMT3A* in macrophages, particularly in omental tissue, supports the hypothesis of epigenetic reprogramming contributing to the development of insulin resistance, as macrophage-driven inflammation is a hallmark of IR-associated adipose remodeling.

The m^5^C readers *MBD3, UNG, SMUG1, NEIL1, NTHL1, MBD4* and *ZBTB33* show higher expression in OVAT compared to SAT. *TDG, YBX1, MBD2* and *UHRF1* showed higher expression levels in SAT compared to OVAT. *NTHL1*, primarily known for its role in DNA repair, has also been identified alongside eight other m^5^C readers as part of a specific m^5^C modification signature used to detect early acute myocardial infarction (34, 35). Similarly, *TDG* has been proposed as a diagnostic m^5^C-associated marker in idiopathic pulmonary fibrosis (36). Additionally, *NEIL1*, a key component of the base excision repair pathway essential for genomic integrity, is also implicated in various human diseases. Defects in *NEIL1* have been associated with increased risks of cancer, diabetes and obesity (37). In the weight-loss two-step bariatric surgery cohort, the reader *YBX1, DNMT1*, writer *NSUN2* and the eraser *TET3* were the only regulators showing a significant differential gene expression between pre-and post-surgery timepoints and were observed downregulated following surgery. This data may hint at a potential impact of weight loss on the expression of these genes and the downregulation of *YBX1* could result in decreased levels of its target proteins as it is known to actively stabilize mRNA through directly binding to m^5^C (38). However, no causative conclusions are possible due to the lack of functional validation in our study. Importantly, given that our cross-sectional cohort comprised only a limited number of non-obese individuals, our comparisons between obese vs non-obese individuals showing in general lower expression levels for the m^5^C machinery in subjects with obesity cannot be overestimated, and more data and larger cohorts are warranted in the future to draw any definite conclusion.

Furthermore, in the MHUO cohort *MBD2* had a lower expression in IR individuals in OVAT compared to the IS counterparts. Given that *MBD2* was predominantly expressed in adipocytes and mesothelial cells in the snucSeq data, this downregulation may reflect a loss of epigenetic repression mechanisms in key stromal compartments. Such a reduction could lead to increased transcription of pro-inflammatory or fibrotic genes, contributing to the dysfunctional remodeling of visceral fat observed in insulin resistance. These findings suggest a potential protective role of *MBD2* in maintaining adipose tissue homeostasis, particularly in visceral depots.

Moreover, we observed higher expression levels in SAT compared to OVAT for the erasers *TET1, TET2*, and *TET3*, while the expression is lower across tissue depots among subjects with obesity. This may potentially hint at a general reduction of the eraser activity suggesting the possibility that individuals with normal weight have lower m^5^C levels compared to obese patients. The TET family is known to be involved in demethylation of both DNA and RNA m^5^C modification, in addition to their ability to yield 5-hydroxymethylcytosine (5hmC), 5-formylcytosine (5fC) and 5-carboxycytosine (5caC) through oxidation of m^5^C in DNA (39, 40). *TET2* has previously been identified as an antiadipogenic demethylase in adipocyte differentiation in 3T3-L1 cells (41). Additionally, *TET3* expression was decreased in SAT following weight loss. These findings are supported by Jung et al. (2023), who showed that *TET3* is important for white adipose tissue development and expansion. Our results from the CSC cohort are in line with this, as we observed a higher *TET3* expression in SAT (42). Jung et al. (2023), further proposed that *TET3* regulates adipogenesis and diet-induced tissue expansion by modulating DNA methylation at important target genes in adipose precursor cells (42). Lastly, due to the involvement of the TET family in multiple forms of modification in DNA and RNA the results should be carefully interpreted as future validation studies are needed to confirm m^5^C methylation levels in the two depots investigated (39, 40).

Collectively, these results demonstrate a distinct depot- and cell-type-specific pattern of m^5^C regulator expression, with omental macrophages showing particularly elevated expression levels. This suggests a potential epigenetic axis through which visceral adipose tissue may contribute to insulin resistance in morbid obesity.

### Clinical variables important for obesity correlate with m^5^C regulators

We find expression of multiple m^5^C regulators correlated with several clinical traits related to obesity and metabolism. In the CSC cohort, anthropometric traits like body fat were significantly correlated with a multitude of genes in both OVAT and SAT. This correlation was more widespread across several regulators in SAT compared to OVAT. Interestingly, *NSUN2* was found to be negatively correlated with body fat for both depots. In line with this, we observed lower *NSUN2* gene expression post-surgery and among subjects with obesity across both adipose tissue depots. These results align with the findings from Liu et al. (2021) showing that overexpression of *NSUN2* corresponds to lower adipogenesis (19). In SAT, two additional *NSUN* family members (*NSUN4* and *NSUN6*) are negatively correlated to body fat. *NSUN4* have been linked to recruiting the degrading machinery for dsRNA in mitochondria to inhibit activation of the immune response due to faulty mitochondrial action (43). This may suggest a potential link between *NSUN4* expression and the increase of immune cells in obesity. Furthermore, the three reader proteins *NEIL1, NTHL1, SMUG1* and the writer *DNMT1* all had a strong positive correlation in SAT with percentage body fat. The reader proteins are mainly known to be involved in the DNA base excision repair pathway, an important mechanism to be functional in an active depot (34). Moreover, in addition we observed strong correlations of multiple m^5^C regulators with mean and maximum adipocyte size suggesting a potential functional mechanism linking the m^5^C machinery to adipose biology. Furthermore, gene expression of multiple m^5^C regulators is correlated with important metabolic variables such as fasting plasma glucose and HOMA-IR across depots. However, only the SAT depot showed positive correlations for the three m^5^C readers *MBD2, MECP2* and *UHRF2* with adiponectin expression. Adiponectin is an adipokine with insulin-sensitizing properties and mainly originates from the SAT depot (44).

Although this is to our knowledge the first study exploring the relationship of m^5^C regulator gene expression and clinical variables related to obesity, our study has several limitations. First, two of the cohorts (BSC and MHUO) have relatively small sample size, which may limit the statistical power and generalizability of the findings described here. Second, the number of subjects with normal weight is relatively low in our cross-sectional cohort and our observed findings need to be interpreted with caution. Third, some of the genes identified by Song et al. (2022) to be members of the m^5^C regulating machinery, are also described as DNA m^5^C regulators (such as DNMT family and MBD family). Therefore, the observed correlation of gene expression with clinical traits may concern both DNA methylation and RNA methylation and should be carefully interpreted. Moreover, other genes may be involved in the regulation of RNA m^5^C, that were not included in the gene set identified by Song et al. (2022) and are therefore not analyzed in our study. Lastly, more functional data are therefore warranted to confirm or reject our results.

## Conclusion

In conclusion, this study is, to our knowledge, the first to establish a link between gene expression profiles of m^5^C regulators and clinical traits related to human obesity. Our results demonstrate that 5-methylcytosine regulators are expressed in an adipose tissue depot-specific manner and that gene expression levels in the different fat depots are differentially correlated with important clinical variables.

## Data Availability

Bulk RNA and snRNA-sequencing data from the LOBB reported in this study cannot be deposited in a public repository due to restrictions by patient consent. These restrictions are due to local data protection regulation in the written informed consent form tissue donors signed before taking part in the study. Access to human adipose tissue biobank data is regulated by the LOBB steering committee. Use of data is strictly limited to research purposes and not intended for commercialization. To request access, contact Matthias Blüher (matthias.blueher@medizin.uni-leipzig.de) or Anne Hoffmann (anne.hoffmann@helmholtz-munich.de). Web application links to explore the datasets from the MHUO cohort are available on GitHub (https://github.com/WolfrumLab/MHUO/).
