# Supplementary figures and images for: Regulators of RNA m5C methylation are adipose tissue depot-specific expressed and correlate with clinical variables of obesity in humans
Source: Front Endocrinol (Lausanne). 2025 Sep 15;16:1647477. doi: 10.3389/fendo.2025.1647477 (PMC12477048; doi:10.3389/fendo.2025.1647477)

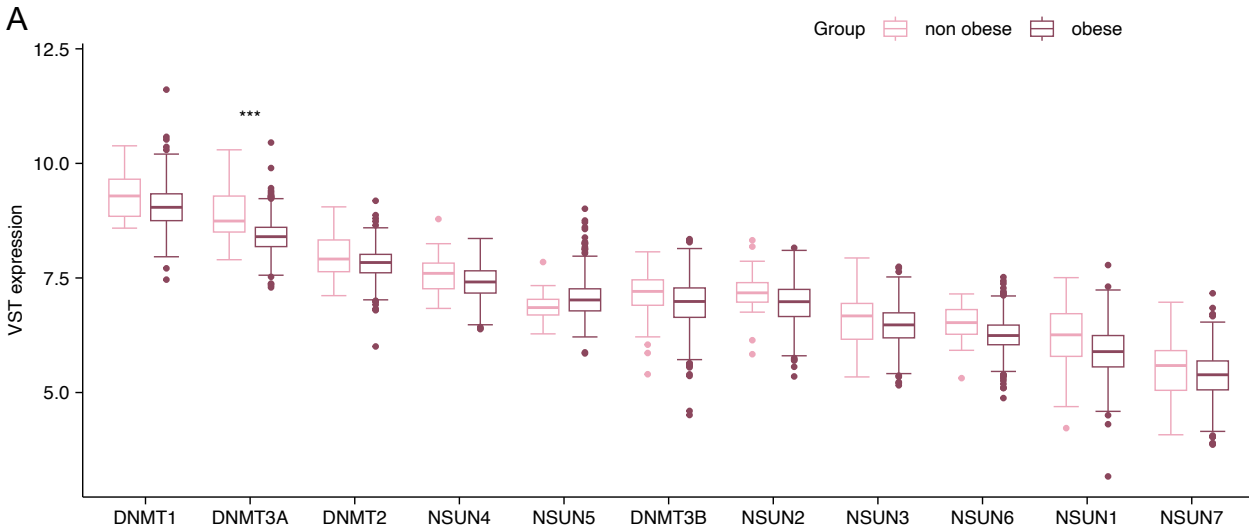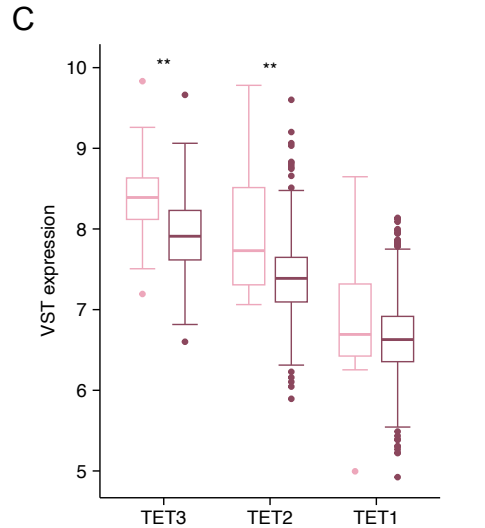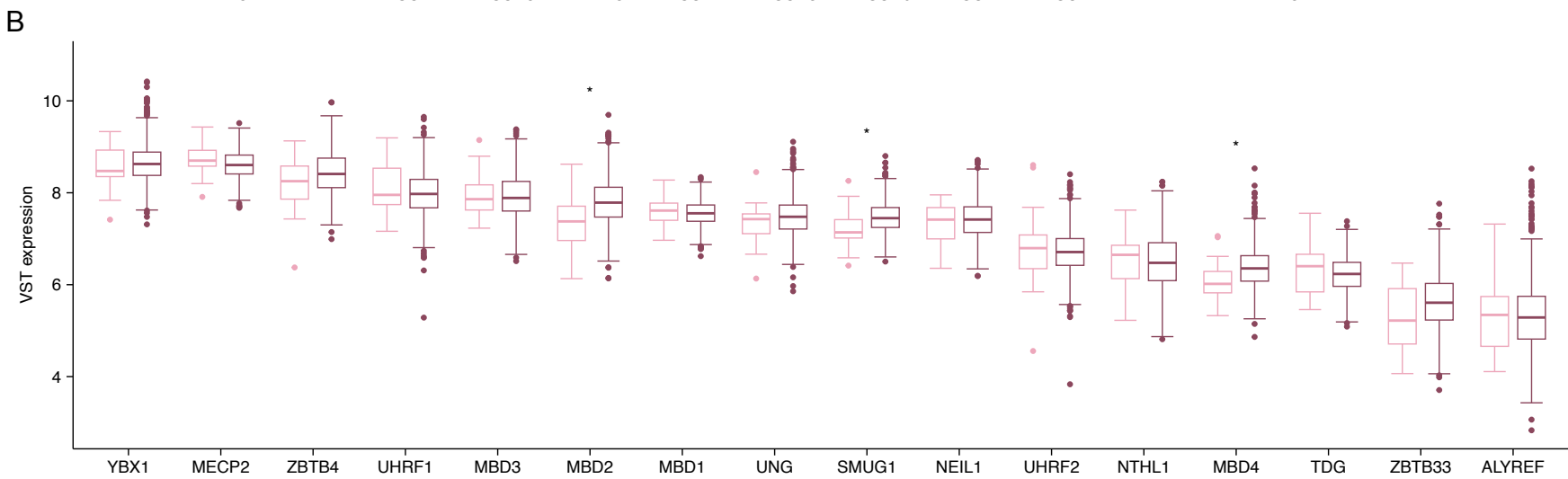

Supplement: Supplementary file 1 [file Supplementaryfile1.pdf]

**A**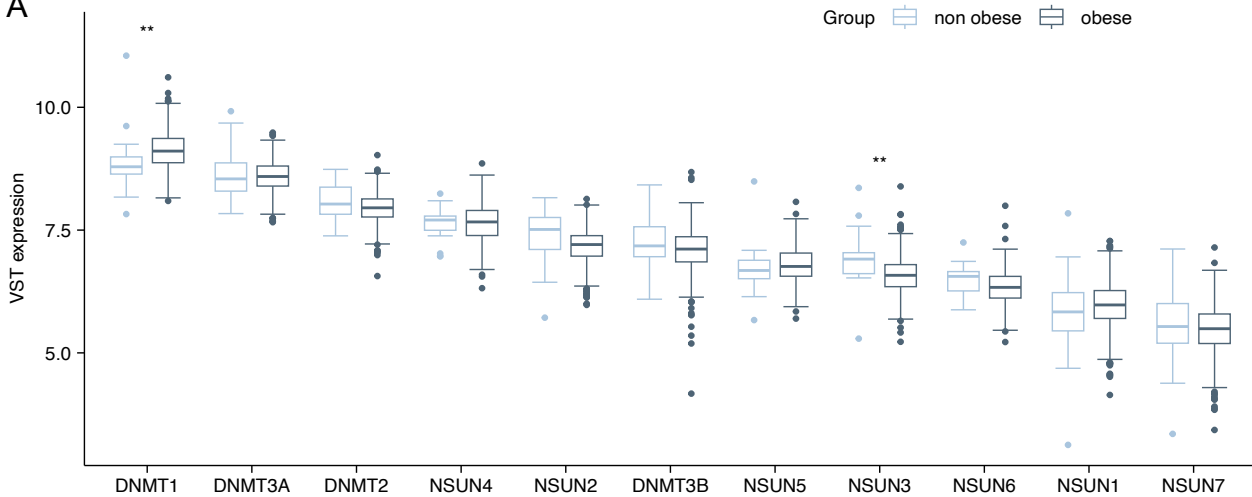**C**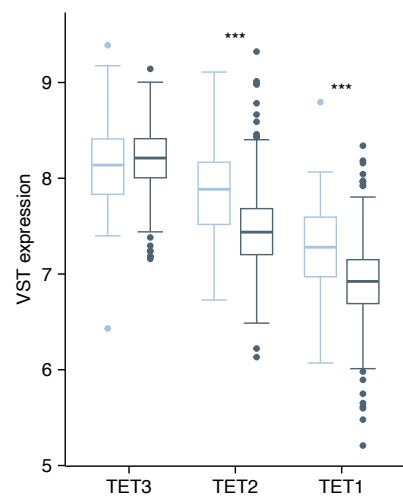**B**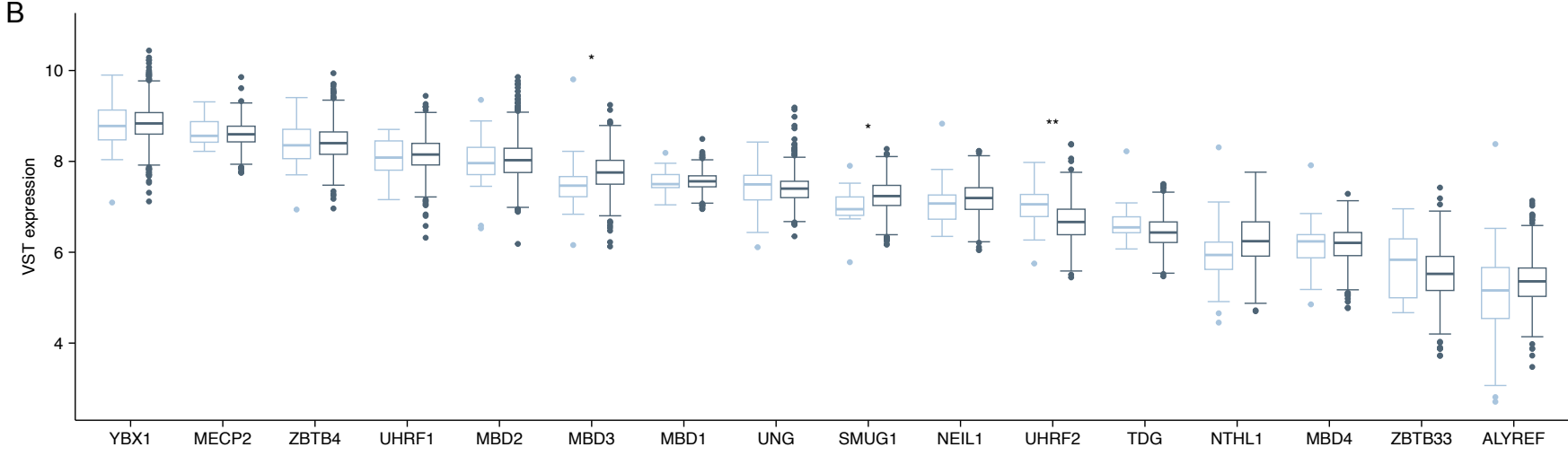

Supplement: Supplementary file 2 [file Supplementaryfile2.pdf]
